# Appendicitis Hospitalization Care Costs Among Patients With Delayed Diagnosis of Appendicitis

**DOI:** 10.1001/jamanetworkopen.2024.6721

**Published:** 2024-04-15

**Authors:** Dinushi A. Kulasekere, Regina Royan, Ying Shan, Ana M. Reyes, Arielle C. Thomas, Alexander L. Lundberg, Joe M. Feinglass, Anne M. Stey

**Affiliations:** 1 Feinberg School of Medicine, Northwestern University, Chicago, Illinois; 2 Department of Emergency Medicine, University of Michigan, Ann Arbor; 3 Department of Surgery, Feinberg School of Medicine, Northwestern University, Chicago, Illinois; 4 Department of Surgery, University of Miami/Jackson Memorial Hospital, Miami, Florida; 5 Department of Surgery, Medical College of Wisconsin, Milwaukee; 6 Department of Medicine, Feinberg School of Medicine, Northwestern University, Chicago, Illinois; 7 Department of Emergency Medicine, Feinberg School of Medicine, Northwestern University, Chicago, Illinois

## Abstract

**Importance:**

Delayed appendicitis diagnosis is associated with worse outcomes. Appendicitis hospital care costs associated with delayed diagnosis are unknown.

**Objective:**

To determine whether delayed appendicitis diagnosis was associated with increased appendicitis hospital care costs.

**Design, Setting, and Participants:**

This cohort study used data from patients receiving an appendectomy aged 18 to 64 years in 5 states (Florida, Maryland, Massachusetts, New York, Wisconsin) that were captured in the Healthcare Cost and Utilization Project State Inpatient and Emergency Department databases for the years 2016 and 2017 with no additional follow-up. Data were analyzed January through April 2023.

**Exposures:**

Delayed diagnosis was defined as a previous emergency department or inpatient hospital encounter with an abdominal diagnosis other than appendicitis, and no intervention 7 days prior to appendectomy encounter.

**Main Outcomes and Measures:**

The main outcome was appendicitis hospital care costs. This was calculated from aggregated charges of encounters 7 days prior to appendectomy, the appendectomy encounter, and 30 days postoperatively. Cost-to-charge ratios were applied to charges to obtain costs, which were then adjusted for wage index, inflation to 2022 US dollar, and with extreme outliers winsorized. A multivariable Poisson regression estimated appendicitis hospital care costs associated with a delayed diagnosis while controlling for age, sex, race and ethnicity, insurance status, care discontinuity, income quartile, hospital size, teaching status, medical school affiliation, percentage of Black and Hispanic patient discharges, core-based statistical area, and state.

**Results:**

There were 76 183 patients (38 939 female [51.1%]; 2192 Asian or Pacific Islander [2.9%], 14 132 Hispanic [18.5%], 8195 non-Hispanic Black [10.8%], 46 949 non-Hispanic White [61.6%]) underwent appendectomy, and 2045 (2.7%) had a delayed diagnosis. Delayed diagnosis patients had median (IQR) unadjusted cost of $11 099 ($6752-$17 740) compared with $9177 ($5575-$14 481) for nondelayed (*P* < .001). Patients with delayed diagnosis had 1.23 times (95% CI, 1.16-1.28 times) adjusted increased appendicitis hospital care costs. The mean marginal cost of delayed diagnosis was $2712 (95% CI, $2083-$3342). Even controlling for delayed diagnosis, non-Hispanic Black patients had 1.22 times (95% CI, 1.17-1.28 times) the adjusted increased appendicitis hospital care costs compared with non-Hispanic White patients.

**Conclusions and Relevance:**

In this cohort study, delayed diagnosis of appendicitis was associated with increased hospital care costs.

## Key Points

**Question:**

How is delayed diagnosis of appendicitis associated with appendicitis hospital care costs?

**Findings:**

In this cohort study of 76 183 patients aged 18 to 64 years who underwent appendectomy in 5 states from 2016 to 2017, delayed diagnosis was associated with a 23% adjusted increase in appendicitis hospital care costs adjusting for age, sex, race and ethnicity, insurance status, care discontinuity, income quartile, hospital size, teaching status, medical school affiliation, percentage of Black and Hispanic patient discharges, core-based statistical area, and state.

**Meaning:**

These results suggest that addressing delayed diagnosis among adult patients may help reduce appendicitis hospital care costs.

## Teaser

This cohort study of patients receiving appendectomies in 5 US states examines the association of delayed diagnosis of appendicitis with increased hospital care costs.

## Introduction

Delayed appendicitis diagnosis has been well documented among both adult and pediatric populations. Delayed diagnoses have resulted in increased appendectomy length of stay and 30-day readmissions.^1,2,3^ And while delayed diagnosis has been commonly observed among non-Hispanic Black patients, hospitals that serve a higher proportion of non-Hispanic Black patients have received lower payer reimbursement for care, exacerbating disparities.^4^ Previous single-site studies have found increased costs, longer lengths of stay, and higher rates of complications among non-Hispanic Black patients.^5,6,7^ Black children with appendicitis have higher care costs and higher postoperative complication rates.^8^ One study found increased total hospital encounter cost among children with longer time to appendectomy, and longer appendectomy duration.^9^ However, it is unclear whether increased costs were associated with delayed appendicitis diagnosis or operational delays to appendectomy. There is a research gap in whether delayed appendicitis diagnosis is associated with increased appendicitis hospital care costs.

This study sought to quantify the differences in appendicitis hospital care costs associated with delayed appendicitis diagnosis. The first aim was to determine whether delayed diagnosis was associated with increased appendicitis hospital care costs. The second aim was to identify how median aggregated appendicitis hospital care costs compared between non-Hispanic Black patients and non-Hispanic White patients after controlling for delayed diagnosis. The third aim was to identify how median aggregated appendicitis hospital care costs compared between hospitals that served mostly Black and Hispanic patients compared with hospitals that served few Black and Hispanic patients. The hypothesis was that delayed diagnosis would be associated with increased adjusted appendicitis hospital care costs even after controlling for patient and hospital variables.

## Methods

### Study Design

The Northwestern University institutional review board approved the study. A waiver of informed consent was obtained due to the deidentified nature of the dataset. The study reporting in this manuscript followed the Strengthening the Reporting of Observational Studies in Epidemiology (STROBE) reporting guidelines.

### Data Sources

Patient records from the Healthcare Cost and Utilization Project’s (HCUP) State Inpatient Database (SID) and State Emergency Department Databases (SEDD) during the years 2016 and 2017 were examined in the year 2022. These all-payer claims data offer the most comprehensive capture of all hospital encounters during the study period for patients of all ages.^10^ Five states (Florida, Maryland, Massachusetts, New York, and Wisconsin) that included the VisitLink identifier were selected to link patient visits between the SID and SEDD databases. Hospital characteristics were obtained from the American Hospital Association Survey and were linked to the patient encounters by the American Hospital Association hospital identifier number.^11^

### Sample Selection

The sample consisted of patients aged 18 to 64 years who had undergone surgical treatment of their appendicitis with an open or laparoscopic appendectomy (eFigure in Supplement 1). The population was defined based on surgical treatment because most patients with appendicitis were treated surgically in 2016 and 2017.^12,13^ These patients were identified in SID using the *International Classification of Diseases, Tenth Revision, Procedure Coding System* (*ICD-10-PCS*) codes for excision, resection, extraction, extirpation, and drainage of the appendix (eTable 1 in Supplement 1). These patients were identified using the *Current Procedural Terminology* (*CPT*) codes for open appendectomy, laparoscopic appendectomy, appendectomy for ruptured appendicitis, and drainage of appendiceal abscess (eTable 2 in Supplement 1). Patients over age 64 years were excluded because more comorbidities may reduce likelihood of initial surgical management. Patients under age 18 years were excluded because appendicitis hospitals care costs have already been studied among children.

### Primary Exposure

The primary exposure was delayed appendicitis diagnosis as a dichotomous variable. It was defined as a previous emergency department or inpatient hospital encounter with an abdominal diagnosis other than appendicitis, and no intervention 7 days prior to subsequent appendectomy encounter.^1,2,3,14^ The timing variable (coded as DaysToEvent) was created with the appendectomy encounter day as the day 0. This variable allowed determination of whether a patient had an emergency department or inpatient hospital encounter in the 7 days preceding their appendectomy. This variable was also used to identify when there were subsequent emergency department or inpatient encounters 30 days following the appendectomy encounter. These postoperative encounters were assumed to indicate additional hospital-based appendicitis postoperative care.^15^

### Primary Outcome

The primary outcome of interest for this study was aggregated appendicitis hospital care costs. The hospital or clinician perspective was used in this economic analysis. The hospital perspective is the most appropriate perspective because hospitals bear the greatest cost burden of revisits, prolonged length of stay, and readmission. Furthermore, hospitals are uniquely able to create and implement interventions to address delayed diagnosis.^16^ The aggregated appendicitis hospital care cost variable combined the cost for all encounters within the 7-day preoperative period before appendectomy, the appendectomy encounter, and all encounters within the 30-day postoperative period following appendectomy. The hospital charge associated with each encounter was multiplied by a hospital- and year-specific cost-to-charge ratio to calculate encounter cost.^17^ All cost-to-charge ratios and calculated costs were winsorized to the first and 99th percentile to adjust for extreme outliers.^18,19^ The encounter-level cost was subsequently adjusted for wage index, or cost of living, and inflation to 2022 US dollars.^20^ The cost of living adjustment was based on a Center for Medicare and Medicaid Services annual wage index linked at the county level using Social Security Administration county codes.^21^

### Patient Characteristics

Patient characteristics included were age, sex, race and ethnicity, insurance status, and zip code median household income. Age was defined into 4 categories including ages 18-24, 25-34, 45-54, and 55-64 years, with 18 to 24 years as the reference category. Sex was defined as a binary variable, with female as the reference category. Race and ethnicity categories were defined by HCUP, which contains coding for this variable as provided by the hospitals. This variable was defined as Asian or Pacific Islander, Hispanic, non-Hispanic Black, non-Hispanic White, and other, which included groups (eg, American Indian or Alaska Native) with relatively small populations in the sample (less than 1%). Non-Hispanic White served as the reference category for the analyses. Insurance status included Medicare, Medicaid, private insurance, and other. Private insurance served as a reference for the analyses. Insurance defined as other included self-pay, no charge, and other as indicated by the HCUP data set. Income categories were separated into quartiles based on the median household income for each patient zip code. In the year 2016, Q1 was $1 to $42,999, Q2 was $43 000 to $53 999, Q3 was $54 000 to $70 999, and Q4 was greater than or equal to $71 000. In the year 2017, Q1 was $1 to $43 999, Q2 was $44 000 to $55 999, Q3 was $56 000 to $73 999, and Q4 was greater than or equal to $74 000. A new dichotomous variable called care discontinuity was created. This dichotomous variable was set to 1 if a patient with delayed diagnosis had their initial encounter at a hospital different from the hospital where they had their subsequent appendectomy.

### Hospital Characteristics

Hospital characteristics included in the study were bed size, teaching status, medical school affiliation, core-based statistical area (CBSA) type, trauma center designation level, state, and hospital percentage of Black and Hispanic patient discharges. Bed size was separated into 3 categories including small (<100 beds), medium (100-499 beds), and large (≥500 beds) with large as reference. Teaching status (teaching, nonteaching), medical school affiliation (affiliated, nonaffiliated) and CBSA type (urban, rural) were all binary variables. Trauma center designation level had 4 categories—levels I, II, III, and IV/nontrauma—where level I was the reference and designated the most highly resourced trauma centers. The hospital percentage was calculated for each hospital each year by taking the total number of non-Hispanic Black and Hispanic discharges for all encounters with any diagnosis and dividing by the total number of all race and ethnicity discharges for all encounters with any diagnosis. Hospitals were then split into quartiles based on whether less than 25%, 25% to 49.99%, 50% to 74.99%, and 75% or more of their discharges were for non-Hispanic Black or Hispanic patients. Percentage of Black and Hispanic patient discharges were included in the model to assess differences in cost for hospitals that serve higher proportions of minority populations.^22,23,24^

### Statistical Analysis

Patient and hospital-level characteristics in the delayed diagnosis cohort and nondelayed diagnosis cohort were compared using a Pearson χ^2^ test. Median aggregated appendicitis hospital care costs by patient and hospital characteristics were calculated for the overall cohort, delayed diagnosis cohort, and nondelayed diagnosis cohort. The Wilcoxon rank-sum test with a Bonferroni correction was used to test for significant differences in unadjusted median cost. A multivariable Poisson regression model was used to estimate the adjusted increased cost of hospital care associated with delayed diagnosis of appendicitis while controlling for patient age, sex, race and ethnicity, insurance status, median income quartile by zip code, hospital bed size, teaching status, medical school affiliation, hospital CBSA type, percentage of Black and Hispanic patient discharges, and state with hospital-clustered robust standard errors.^25^ Poisson regression was chosen because the distribution of cost was highly right-skewed.^26,27^ Poisson regression can be used with a continuous dependent variable, as coefficient estimates are consistent provided the conditional mean component of the model is correctly specified.^28^ The 95% confidence intervals around the exponentiated coefficient estimates were calculated for each variable category. All statistical tests were considered significant at *P* < .05. All statistical analyses were performed using Stata 17 (StataCorp LLC) and SAS version 9.4 (SAS Institute).

## Results

The final cohort consisted of 76 183 unique patients who received an appendectomy, of whom 2045 (2.7%) had a delayed diagnosis. The cohort included 38 939 female (51.1%) and 37 244 male (48.9%) patients (Table 1). By age, the largest cohort of patients were ages 25 to 34 years (18 399 [24.1%]) while 13 626 patients (17.9%) were aged 55 to 64 years. The cohort comprised 2192 Asian or Pacific Islander (2.9%), 14 132 Hispanic (18.5%), 8195 non-Hispanic Black (10.8%), and 46 949 non-Hispanic White patients (61.6%). A total of 47 812 patients (62.8%) were privately insured, while 15 157 (19.9%) were covered by Medicaid, and 3038 (4.0%) were covered by Medicare disability.

**Table 1.** Characteristics of Patients Undergoing Appendectomy in Massachusetts, Florida, Maryland, New York, and Wisconsin, 2016-2017

Characteristic	Patients, No. (%)			Pearson χ^2^ *P* value
	Total (n = 76 183)	Delayed diagnosis (n = 2045)	Nondelayed diagnosis (n = 74 138)	
Age				
18-24 y	13 996 (18.4)	477 (23.3)	13 519 (18.2)	<.001
25-34 y	18 399 (24.1)	621 (30.4)	17 778 (24.0)	
35-44 y	14 939 (19.6)	387 (18.9)	14 552 (19.6)	
45-54 y	15 223 (20.0)	328 (16.0)	14 895 (20.1)	
55-64 y	13 626 (17.9)	232 (11.3)	13 394 (18.1)	
Sex				
Male	37 244 (48.9)	753 (36.8)	36 491 (49.2)	<.001
Female	38 939 (51.1)	1292 (63.2)	37 647 (50.8)	
Race and ethnicity				
Asian or Pacific Islander	2192 (2.9)	43 (2.1)	2149 (2.9)	<.001
Hispanic	14 132 (18.5)	355 (17.4)	13 777 (18.6)	
Non-Hispanic Black	8195 (10.8)	319 (15.6)	7876 (10.6)	
Non-Hispanic White	46 949 (61.6)	1226 (60.0)	45 723 (61.7)	
Other^a^	4715 (6.2)	102 (4.9)	4613 (6.2)	
Insurance status				
Medicare	3038 (4.0)	91 (4.4)	2947 (4.0)	<.001
Medicaid	15 157 (19.9)	554 (27.1)	14 603 (19.7)	
Private insurance	47 812 (62.8)	1088 (53.2)	46 724 (63.0)	
Other^b^	10 176 (13.3)	312 (15.3)	9864 (13.3)	
Zip code median income quartile^c^				
1	18 437 (24.2)	610 (29.8)	17 827 (24.1)	<.001
2	19 131 (25.1)	589 (28.8)	18 542 (25.0)	
3	19 670 (25.8)	460 (22.5)	19 210 (25.9)	
4	18 945 (24.9)	386 (18.9)	18 559 (25.0)	
Care discontinuity^d^				
Yes	NA	903 (44.2)	NA	<.001
No	NA	1142 (55.8)	74 138 (100.0)	
Perforated appendicitis				
Yes	7728 (10.1)	273 (13.4)	7455 (10.1)	<.001
No	68 455 (89.9)	1772 (86.6)	66 683 (89.9)	

^a^ Other includes groups (eg, American Indian or Alaska Native) with relatively small populations in the sample (less than 1%).

^b^ Other includes self-pay, no charge, and any other insurance.

^c^ 2016 (quartile 1, $1-$42 999; quartile 2, $43 000-$53 999; quartile 3, $54 000-$70 999; quartile 4, ≥$71 000); 2017 (quartile 1, $1-$43 999; quartile 2, $44 000-$55 999; quartile 3, $56 000-$73 999; quartile 4, ≥$74 000).

^d^ Appendectomy performed at a hospital different from initial presenting hospital.

### Delayed Diagnosis Cohort

Female patients made up a higher proportion of the delayed diagnosis cohort, 63.2% (1292 of 2045 patients) compared with 50.8% (37 647 of 74 138 patients) in the nondelayed cohort. Non-Hispanic Black patients also made up a higher proportion of the delayed diagnosis cohort, 15.6% (319 of 2045 patients) compared with 10.6% (7876 of 74 138 patients) in the nondelayed cohort. Among all racial and ethnic categories, non-Hispanic Black patients had the highest rate of delayed diagnosis 3.9% (319 of 8195), compared with 2.6% (1226 of 46 949) among non-Hispanic White patients, 2.5% (355 of 14 132) among Hispanic patients, 2.2% (102 of 4715) among patients of other race and ethnicity, and 2.0% (43 of 2192) among Asian patients. Among all 2045 patients with delayed diagnosis, 903 (44.5%) had their initial encounter at a hospital different from the hospital where they received an appendectomy or care discontinuity.

### Hospital Characteristics

Hospital characteristics were similar between the delayed cohort and the nondelayed cohort with the exception of medical school affiliation and percentage of Black and Hispanic patient discharges (Table 2). The delayed cohort had a larger proportion of patients treated at nonaffiliated hospitals (930 of 2045 [45.5%]) compared with patients in the nondelayed cohort (30 849 of 74 138 [41.6%]) (*P* < .001). The delayed cohort had a higher proportion of patients treated at hospitals with less than 25% Black or Hispanic patient discharges (797 of 2045 [39.0%]) compared with the nondelayed cohort (27 096 of 74 138 [36.6%]) (*P* = .007).

**Table 2.** Hospital Characteristics for Appendectomies Performed in Massachusetts, Florida, Maryland, New York, and Wisconsin, 2016-2017

Characteristic	Patients, No. (%)			Pearson χ^2^ *P* value
	Total (n = 76 183)	Delayed diagnosis (n = 2045)	Nondelayed diagnosis (n = 74 138)	
Bed size				
Small (<100 beds)	6412 (8.4)	191 (9.3)	6221 (8.4)	.31
Medium (100-499 beds)	46 378 (60.9)	1229 (60.1)	45 149 (60.9)	
Large (≥500 beds)	23 393 (30.7)	625 (30.6)	22 768 (30.7)	
Teaching status				
Teaching	18 267 (24.0)	476 (23.3)	17 791 (24.0)	.45
Nonteaching	57 916 (76.0)	1569 (76.7)	56 347 (76.0)	
Medical school affiliation				
Affiliated	44 404 (58.3)	1115 (54.5)	43 289 (58.4)	<.001
Nonaffiliated	31 779 (41.7)	930 (45.5)	30 849 (41.6)	
Core-based statistical area				
Urban	75 696 (99.4)	2030 (99.3)	73 666 (99.4)	.59
Rural	487 (0.6)	15 (0.7)	472 (0.6)	
Proportion of Black and Hispanic patients				
<25%	27 893 (36.6)	797 (39.0)	27 096 (36.6)	.007
25%-49.99%	28 686 (37.7)	788 (38.5)	27 898 (37.6)	
50%-74.99%	14 204 (18.6)	328 (16.2)	13 872 (18.7)	
≥75%	5400 (7.1)	128 (6.3)	5272 (7.1)	
Trauma center level				
I (Regional resource center)	13 595 (17.8)	356 (17.4)	13 239 (17.9)	.52
II (Community trauma center)	10 683 (14.0)	290 (14.2)	10 393 (14.0)	
III (Rural trauma center)	6753 (8.9)	200 (9.8)	6553 (8.8)	
IV (Other trauma center)	541 (0.7)	11 (0.5)	530 (0.7)	
V (Not recorded)	44 611 (58.6)	1188 (58.1)	43 423 (58.6)	
State				
Florida	32 286 (42.4)	934 (45.7)	31 352 (42.3)	<.001
Maryland	9241 (12.1)	212 (10.3)	9029 (12.2)	
Massachusetts	3487 (4.6)	109 (5.3)	3378 (4.5)	
New York	21 844 (28.7)	490 (24.0)	21 354 (28.8)	
Wisconsin	9325 (12.2)	300 (14.7)	9029 (12.2)	

### Unadjusted Appendicitis Hospital Care Costs

In the 5 states we considered in 2016 to 2017, the median (IQR) unadjusted cost of nondelayed appendicitis was $9177 ($5575-$14 481). Patients with delayed diagnosis had higher median cost across all age groups, sex, race and ethnicity, insurance status, and income quartile compared with the nondelayed group (Table 3). Delayed diagnosis had a significantly higher median (IQR) unadjusted cost of $11 099 ($6752-$17 740). Notably, non-Hispanic Black patients with delayed diagnosis had a higher unadjusted median (IQR) cost ($13 027 [$8004-$19 947]) compared with non-Hispanic Black patients with nondelayed diagnosis ($10 650 [$6471-$17 074]). If patients experienced care discontinuity, they had a significantly higher median cost of $11 794 ($6858-$18 431) compared with $10 657 ($6597-$17 165) among patients with delayed diagnosis that did not experience discontinuity. Patients with delayed diagnosis generally showed higher cost across all hospital characteristics compared with the nondelayed cohort (Table 4).

**Table 3.** Median Aggregated Cost of Care for Patients Undergoing Appendectomy With Delayed Diagnosis Stratified by Demographic and Clinical Characteristics^a^

Characteristic	Median cost (IQR), $			Wilcoxon rank sum *P* value
	Total (n = 76 183)	Delayed diagnosis (n = 2045)	Nondelayed diagnosis (n = 74 138)	
Total	9214 (5607-14 569)	11 099 (6752-17 740)	9177 (5575-14 481)	<.001
Age				
18-24 y	7604 (4952-11 502)	9222 (6016-14 606)	7559 (4917-11 436)	<.001
25-34 y	8226 (5166-12 344)	10 504 (6417-15 733)	8169 (5130-12 245)	<.001
35-44 y	9026 (5528-14 005)	11 790 (7342-17 828)	8963 (5490-13 907)	<.001
45-54 y	10 476 (6232-16 905)	13 915 (7728-23 652)	10 415 (6213-16 786)	<.001
55-64 y	12 245 (7444-20 871)	14 535 (9336-28 742)	12 207 (7425-20 799)	<.001
Sex				
Male	9051 (5485-14 240)	11 124 (6842-17 599)	9005 (5465-14,59)	<.001
Female	9386 (5717-14 876)	11 075 (6650-17 833)	9341 (5686-14 784)	<.001
Race and ethnicity				
Asian or Pacific Islander	10 277 (6887-15 062)	16 429 (9685-25 926)	10 245 (6817-14 907)	<.001
Hispanic	9517 (6105-13 771)	11 093 (7057-16 451)	9485 (6079-13 663)	<.001
Non-Hispanic Black	10 726 (6540-17 161)	13 027 (8004-19 947)	10 650 (6471-17 074)	<.001
Non-Hispanic white	8571 (5206-14 083)	10 575 (6373-17 165)	8522 (5180-13 993)	<.001
Other^b^	11 322 (7357-16 687)	12 250 (6651-20 774)	11 314 (7360-16 659)	.39
Insurance status				
Medicare	14 327 (8882-26 731)	16 489 (8883-35 833)	14 290 (8880-26 641)	.23
Medicaid	10 488 (6526-15 877)	11 469 (6768-18 190)	10 455 (6507-15 763)	<.001
Private insurance	8729 (5361-13 968)	10 877 (6772-17 478)	8684 (5331-13 883)	<.001
Other^c^	8539 (5242-12 807)	10 595 (6288-14 625)	8491 (5216-12 732)	<.001
Zip code median income quartile^d^				
1	9897 (6051-15 300)	11 417 (6879-17 118)	9851 (6033-15 226)	<.001
2	8864 (5359-14 136)	10 831 (6646-18 047)	8806 (5325-13 999)	<.001
3	8793 (5350-13 920)	10 402 (6422-17 588)	8759 (5326-13 840)	<.001
4	9387 (5760-14 950)	11 952 (7329-19 032)	9336 (5732-14 875)	<.001
Care discontinuity^e^				
Yes	NA	11 794 (6858-18 431)	NA	<.001
No	NA	10 657 (6597-17 165)	9177 (5575-14 481)	
Perforated appendicitis				
Yes	14 050 (9922-21 019)	15 755 (11 032-24 831)	13 978 (9904-20 903)	<.001
No	8706 (5360-13 678)	10 502 (6363-16 574)	8668 (5337-13 587)	<.001

Abbreviation: NA, not applicable.

^a^ Includes study cohort of procedures in Massachusetts, Florida, Maryland, New York, and Wisconsin in 2016-2017.

^b^ Other includes groups (eg, American Indian or Alaska Native) with relatively small populations in the sample (less than 1%).

^c^ Other includes self-pay, no charge, and any other insurance.

^d^ 2016 (quartile 1, $1-$42 999; quartile 2, $43 000-$53 999; quartile 3, $54 000-$70 999; quartile 4, ≥$71 000); 2017 (quartile 1, $1-$43 999; quartile 2, $44 000-$55 999; quartile 3, $56 000-$73 999; quartile 4, ≥$74 000).

^e^ Appendectomy performed at a hospital different from initial presenting hospital.

**Table 4.** Median Aggregated Cost of Care for Patients Undergoing Appendectomy With Delayed Diagnosis Stratified by Hospital Characteristics^a^

Characteristic	Median cost (IQR), $			Wilcoxon rank sum *P* value
	Total (n = 76 183)	Delayed diagnosis (n = 2045)	Nondelayed diagnosis (n = 74 138)	
Bed size				
Small (<100 beds)	7860 (5179-12 991)	8947 (5537-17 090)	7830 (5168-12 860)	.007
Medium (100-499 beds)	8554 (5118-13 769)	10 449 (6205-16 468)	8514 (5087-13 676)	<.001
Large (≥500 beds)	10 755 (7178-16 720)	13 034 (8769-21 579)	10 702 (7142-16 613)	<.001
Teaching status				
Teaching	11 636 (8116-18 789)	14 068 (9387-24 297)	11 584 (8092-18 653)	<.001
Nonteaching	8357 (5090-13 448)	10 300 (6177-16 400)	8318 (5065-13 362)	<.001
Medical school affiliation				
Affiliated	10 026 (6127-15 796)	11 794 (7349-19 243)	9985 (6093-15 705)	<.001
Nonaffiliated	8192 (4960-12 938)	10 320 (6069-16 381)	8146 (4927-12 822)	<.001
Core-based statistical area				
Urban	9226 (5614-14 575)	11 117 (6752-17 807)	9188 (5583-14 492)	<.001
Rural	6917 (4725-12 299)	9187 (5955-14 902)	6824 (4698-12 242)	.15
Proportion of Black and Hispanic patients^b^				
<25%	8401 (5163-14 347)	10 632 (6358-17 388)	8361 (5136-14 266)	<.001
25%-49.99%	9242 (5479-14 647)	10 630 (6502-17 432)	9204 (5455-14 577)	<.001
50%-74.99%	10 103 (6320-14 312)	11 693 (8245-17 678)	10 063 (6279-14 210)	<.001
≥75%	10 319 (6784-16 066)	13 734 (8797-23 033)	10 255 (6758-15 946)	<.001
Trauma center level				
I (Regional resource center)	11 339 (8060-18 847)	14 123 (10 108-25 999)	11 278 (8017-18 750)	<.001
II (Community trauma center)	9950 (5727-14 857)	11 291 (6800-17 310)	9907 (5705-14 779)	<.001
III (Rural trauma center)	8729 (5542-14 911)	12 422 (6927-18 949)	8639 (5507-14 791)	<.001
IV (Other trauma center)	7496 (5530-10 453)	13 458 (6295-19 032)	7456 (5485-10 358)	.02
V (Not recorded)	8366 (5039-13 396)	9970 (6006-15 810)	8334 (5017-13 330)	<.001
State				
Florida	8009 (4655-11 997)	9793 (5770-14 742)	7972 (4631-11 912)	<.001
Maryland	6683 (4922-11 178)	8916 (6095-14 585)	6637 (4906-11 089)	<.001
Massachusetts	18 349 (13 986-27 564)	21 955 (14 357-37 661)	18 315 (13 986-27 227)	.03
New York	11 710 (8135-17 440)	13 048 (8230-20 537)	11 680 (8134-17 398)	.002
Wisconsin	7956 (5849-13 389)	12 123 (7760-20 271)	7875 (5811-13 138)	<.001

^a^ Includes study cohort of procedures in Massachusetts, Florida, Maryland, New York, and Wisconsin in 2016 to 2017.

^b^ Calculated based on all visits for all causes in 2016 and 2017 in each hospital.

### Adjusted Aggregated Appendicitis Hospital Care Costs

Patients with delayed diagnosis had 1.23 times (95% CI, 1.17-1.29 times) increased adjusted appendicitis hospital care costs after controlling for age, sex, race and ethnicity, insurance status, care discontinuity, income quartile, hospital size, teaching status, medical school affiliation, CBSA type, percentage of Black and Hispanic patient discharges, and state (Table 5). Because our goal was to estimate the preventable costs of delayed diagnosis of appendicitis, we performed additional calculations to estimate the mean marginal cost of delayed diagnosis, which was found to be $2712 (95% CI, $2083-$3342). All age groups had significantly higher costs compared with those aged 18 to 24 years, with the highest increase occurring among those aged 55 to 64 years, at an adjusted increased cost 1.84 times (95% CI, 1.77-1.92 times) higher. Non-Hispanic Black patients had 1.22 times (95% CI, 1.17-1.28 times) increased adjusted cost compared with non-Hispanic White patients even after controlling for delayed diagnosis. Patients insured by Medicare disability had 1.53 times (95% CI, 1.46-1.61 times) increased adjusted cost, while patients insured by Medicaid had 1.16 times (95% CI, 1.12-1.20 times) increased adjusted cost compared with privately insured patients. Hospitals that treated 50% to 74.99% non-Hispanic Black and Hispanic patients had 1.15 times (95% CI 1.04-1.27 times) higher adjusted costs compared with hospitals that treated less than 25% non-Hispanic Black and Hispanic patients. Hospitals treating greater than 75% non-Hispanic Black and Hispanic patients had 1.39 times (95% CI 1.21-1.59 times) higher adjusted cost compared with hospitals that treated less than 25% non-Hispanic Black and Hispanic patients.

**Table 5.** Poisson Regression Model of Aggregated Cost of Care on Delayed Diagnosis for Patients Undergoing Appendectomy Adjusted for Hospital^a^

Characteristic	Delayed diagnosis, estimated increase (95% CI) (n = 2045)	*P* value
Delayed diagnosis	1.23 (1.17-1.29)	<.001
Age		
18-24 y	1 [Reference]	[Reference]
25-34 y	1.09 (1.07-1.12)	<.001
35-44 y	1.27 (1.23-1.31)	<.001
45-54 y	1.52 (1.47-1.58)	<.001
55-64 y	1.84 (1.77-1.92)	<.001
Sex		
Male	1 [Reference]	[Reference]
Female	0.98 (0.95-1.01)	.16
Race and ethnicity		
Non-Hispanic White	1 [Reference]	[Reference]
Asian or Pacific Islander	0.99 (0.93-1.07)	.89
Hispanic	0.96 (0.92-1.00)	.04
Non-Hispanic Black	1.22 (1.17-1.28)	<.001
Other^b^	1.07 (1.00-1.14)	.047
Insurance status		
Private insurance	1 [Reference]	[Reference]
Medicare	1.53 (1.46-1.61)	<.001
Medicaid	1.16 (1.12-1.20)	<.001
Other^c^	1.06 (1.03-1.09)	<.001
Care discontinuity^d^		
No	1 [Reference]	[Reference]
Yes	1.06 (0.98-1.15)	.13
Zip code median income quartile^e^		
4	1 [Reference]	[Reference]
3	0.95 (0.92-0.98)	.001
2	0.95 (0.91-0.99)	.02
1	0.99 (0.94-1.03)	.54
Bed size		
Large (≥500 beds)	1 [Reference]	[Reference]
Medium (100-499 beds)	1.02 (0.87-1.20)	.77
Small (<100 beds)	0.98 (0.83-1.16)	.82
Hospital teaching status		
Nonteaching	1 [Reference]	[Reference]
Teaching	1.39 (1.18-1.64)	<.001
Medical school affiliation		
Nonaffiliated	1 [Reference]	[Reference]
Affiliated	1.00 (0.93-1.07)	.97
CBSA type		
Urban	1 [Reference]	[Reference]
Rural	1.04 (0.85-1.26)	.70
Proportion of Black and Hispanic patients		
<25%	1 [Reference]	[Reference]
25%-49.99%	1.06 (0.96-1.17)	.22
50%-74.99%	1.15 (1.04-1.27)	.009
≥75%	1.39 (1.21-1.59)	<.001
Hospital state		
Massachusetts	1 [Reference]	[Reference]
Florida	0.45 (0.40-0.51)	<.001
Maryland	0.46 (0.39-0.53)	<.001
New York	0.64 (0.56-0.73)	<.001
Wisconsin	0.56 (0.51-0.62)	<.001

Abbreviation: CBSA, core-based statistical area.

^a^ Includes study cohort of procedures in Massachusetts, Florida, Maryland, New York, and Wisconsin in 2016 to 2017 (76 183 patients total).

^b^ Other includes groups (eg, American Indian or Alaska Native) with relatively small populations in the sample (less than 1%).

^c^ Other includes self-pay, no charge, and any other insurance.

^d^ Appendectomy performed at a hospital different from initial presenting hospital.

^e^ 2016 (quartile 1, $1-$42 999; quartile 2, $43 000-$53 999; quartile 3, $54 000-$70 999; quartile 4, ≥$71 000); 2017 (quartile 1, $1-$43 999; quartile 2, $44 000-$55 999; quartile 3, $56 000-$73 999; quartile 4, ≥$74 000).

## Discussion

This study sought to quantify the differences in appendicitis hospital care costs associated with delayed appendicitis diagnosis. This estimate includes perforated appendicitis, as well as any 30-day revisits to give a more realistic cost comparison because patients present with perforated appendicitis for many reasons other than delayed diagnosis.^29,30,31^ Appendicitis is diagnosed for 292 297 patients annually in the US.^32^ A 2.7% rate of delayed diagnosis will impact 7892 patients annually and, given the mean marginal cost difference is $2712.45, this leads to $21 406 655 of preventable cost from poor diagnostic quality every single year. Appendicitis hospital care costs were significantly higher among non-Hispanic Black patients and non-Hispanic White patients after controlling for delayed diagnosis. Delayed diagnosis in addition to leading to worst quality of health and higher costs of care is inequitably distributed, concentrated in racial and ethnic minority populations. Delayed diagnosis is a unique target example of what is commonly described as waste in medical treatment, with poor quality at high cost, as well as a practice that exacerbates inequity of health care access and inequity in health among patients from racial and ethnic minority groups. What we find most alarming is the inequity in how delayed diagnosis is distributed by sex, race and ethnicity, and ethnicity, as well as the increased costs concentrated in minoritized patient populations that result. It is well established that women and patients from racial and ethnic minority populations have more frequent delays in guideline-driven care for acute myocardial infarction, and experience delayed care in injury, stroke, and critical illness.^33,34,35,36,37,38^ Although we cannot be certain in this analysis as to why it happens, prior research suggests that interventions that may help address these inequities in timely diagnosis and care include hiring more women and clinicians from racial and ethnic minority groups in medicine as well as more standardized diagnosis and treatment protocols.^39,40,41,42,43^ These findings were similar to what has been observed in research of surgical disparities.^23,44,45,46^ They also support that reducing delayed diagnosis could reduce overall costs of care. From the perspective of hospital leadership, one way to reduce delayed diagnosis and its potential costs is to work at the institutional level to ensure culturally informed care is provided to patients of all racial and ethnic backgrounds. We could postulate that clinicians are less thorough in their assessment of minoritized patients, resulting in higher rates of delayed diagnosis. However, empirically understanding the mechanism of how minoritized patients experience more delayed diagnosis and higher cost of care warrants further study to better understand the social drivers of health in these populations.

Furthermore, minority surgical care is concentrated within certain hospitals.^22,23,24,47,48,49,50^ Delayed appendicitis diagnoses were less common at hospitals serving greater than 75% of Black and Hispanic patients as previously observed.^1^ Yet, hospitals serving greater than 75% of Black and Hispanic patients had higher appendicitis hospital care costs among both delayed and nondelayed cohorts. Why hospitals serving racial and ethnic minority populations at higher rates were dealing with higher costs is likely multifactorial. Some of these hospitals may be safety-net hospitals with fewer quality improvement resources to facilitate patient throughput.^23,51,52,53,54^ Patients from racial and ethnic minority groups have more health care access barriers, leading to more advanced comorbidities as well as appendicitis at time of emergency department presentation, and thus more complications.^22,23,24,47,48,49,50^ Value-based reimbursement policies penalize these hospitals disproportionately and thereby perpetuate racial and ethnic disparities in health care.^22,52,55,56,57,58^ Furthermore, this study reaffirms that hospitals serving high proportions of minority populations provided high-quality care because they had lower rates of delayed appendicitis diagnosis compared with hospitals that serve relatively very few patients from racial and ethnic minority groups. Delayed diagnosis may be a more equitable quality metric for payers to construct value-based reimbursement policies. This would better reward high-quality performance at hospitals treating more patients from racial and ethnic minority groups, which would help to offset some of the additional cost burden they are facing to improve the overall quality of care instead of differentially penalizing them and worsening health system inequities. Important future studies to add to this work include (1) a qualitative understanding of the diagnostic process for appendicitis and how it differs among racial and ethnic groups; (2) a comprehensive research review with contemporary rates of multi-slice computed tomography use and associated low false positive rates as well as the extremely low complication rates and cost of laparoscopic appendectomy; and (3) a decision analysis to create evidence to support a more standardized diagnostic approach for acute appendicitis.

### Limitations

This study has multiple limitations. First, approximately 10% of patient encounters had missing patient encounter linkage variables, 1% had missing demographic data, and 9% had missing cost data. Although the cohort was large, this may have led to sampling bias. However, 10% is the generally accepted threshold for missingness that requires multiple imputation or other approaches to reduce bias.^59,60^ Second, our analysis does not specifically consider the detailed implications related to the flow of health care dollars, such as the increased costs of using additional imaging to avoid delayed diagnosis or the decreased costs associated with antibiotic treatment. Our data set does not contain the encounter-level data of these variables to empirically answer these questions; however, this is an important component of cost analysis in this population that warrants further study. Third, censoring was a risk of bias. Patients undergoing appendectomy at the end of the year were excluded to limit risk of left or right censoring. Fourth, the VisitLink variable is state specific; therefore preoperative and postoperative encounters that occurred in other states were not captured. As a result, the delayed diagnosis rate as well as total appendicitis hospital care costs may have been underestimated. Fifth, although we did analyze patients with perforated appendicitis, high variability between hospitals, high rates of inaccurate diagnosis coding, and poor capture in diagnostic codes may have prevented accurate estimates of perforation rate.^61,62,63^ Sixth, the use of cost-to-charge ratios to estimate cost is a validated cost analysis technique.^64^ However, there are inherent limitations from estimating surrogate costs from charge data as opposed to having true cost data via cost accounting. Some inaccuracies were addressed by winsorizing extreme outliers, adjusting for inflation 2022 dollar, and adjusting for cost-of-living, but error may persist given the limitations of cost-to-charge ratios. Seventh, delayed appendicitis diagnosis was based on occurrence of an appendicitis procedural intervention and a prior encounter within 7 days with an abdominal complaint. Patients who had nonsurgical management of their appendicitis were not included as antibiotics were not considered standard of care during the study period. A subset of nonsurgically managed appendicitis patients may have experienced delayed diagnosis and have prolonged hospital stays, readmissions, and cost burden. However, nonsurgical management of appendicitis is fairly recent in the US and in 2016 and 2017 was not common in our data set. Future studies assessing delayed diagnosis in patients treated after 2020 would require additional analysis of the cost implications related to nonoperative management, including the growing trend of initial treatment with antibiotics. Finally, no patient-level social drivers of health were known through our current data set. This unmeasured variable bias likely partially explains why Black patients and women experienced higher rates of delayed diagnosis.

## Conclusion

In this cohort study, delayed diagnosis was associated with increased appendicitis hospital care costs. Appendicitis hospital care costs were significantly higher among non-Hispanic Black patients and non-Hispanic White patients after controlling for delayed diagnosis. Appendicitis hospital care costs were higher among hospitals that served greater than 75% Black and Hispanic patients compared with hospitals that served less than 25% Black and Hispanic patients. These results suggest that delayed diagnosis may be a more equitable quality metric for payers to construct value-based reimbursement policies, one that would reward high-quality performance at hospitals that treat a greater proportion of patients from racial and ethnic minority groups, which would in turn help offset some of the cost burden instead of differentially penalizing them and worsening health system inequities.

## Notes

Supplement 1. **eTable 1.** International Classification of Diseases, Tenth Revision, Procedure Coding System (ICD-10-PCS) and *Current Procedural Terminology* (*CPT*) Codes for Appendectomy **eTable 2.** International Classification of Diseases, Tenth Revision, Clinical Modification (ICD-10-CM) Codes for Abdominal Complaints **eFigure.** Flow Diagram of Patient Sample Construction (2016 and 2017)

Supplement 2.
Data Sharing Statement
