# Supplementary material for: Appendicitis Hospitalization Care Costs Among Patients With Delayed Diagnosis of Appendicitis
Source: JAMA Netw Open. 2024 Apr 15;7(4):e246721. doi: 10.1001/jamanetworkopen.2024.6721 (PMC11019393; doi:10.1001/jamanetworkopen.2024.6721)
Supplement: Supplement 1. — eTable 1. International Classification of Diseases, Tenth Revision, Procedure Coding System (ICD-10-PCS) and Current Procedural Terminology (CPT) Codes for Appendectomy eTable 2. International Classification of Diseases, Tenth Revision, Clinical Modification (ICD-10-CM) Codes for Abdominal Complaints eFigure. Flow Diagram of Patient Sample Construction (2016 and 2017) [file jamanetwopen-e246721-s001.pdf]

Supplemental Online Content

Kulasekere DA, Royan R, Shan Y, et al. Appendicitis hospitalization care costs among patients with delayed diagnosis of appendicitis. *JAMA Netw Open.* 2024;7(4):e246721. doi:10.1001/jamanetworkopen.2024.6721

eTable 1. *International Classification of Diseases, Tenth Revision, Procedure Coding System* (*ICD-10-PCS*) and Current Procedural Terminology (*CPT*) Codes for Appendectomy

eTable 2. *International Classification of Diseases, Tenth Revision, Clinical Modification* (*ICD-10-CM*) Codes for Abdominal Complaints

eFigure. Flow Diagram of Patient Sample Construction (2016 and 2017)

This supplemental material has been provided by the authors to give readers additional information about their work.

© 2024 Kulasekere DA et al. *JAMA Network Open.*

eTable 1. ***International Classification of Diseases, Tenth Revision, Procedure Coding System* (*ICD-10-PCS*) and Current Procedural Terminology (CPT) Codes for Appendectomy**

ICD-10-PCS Code	Description	CPT Code	Description
0DBJ0	Excision of Appendix, Open Approach	44900	Drainage of appendiceal abscess
0DBJ3	Excision of Appendix, Percutaneous Approach	44901	Drainage of appendiceal abscess
0DBJ4	Excision of Appendix, Percutaneous Endoscopic Approach	44950	Appendectomy, open approach
0DBJ7	Excision of Appendix, Via Natural or Artificial Opening	44955	Appendectomy when done for indicated purpose at time of other major procedure
0DBJ8	Excision of Appendix, Via Natural or Artificial Opening Endoscopic	44960	Appendectomy for ruptured appendix, with abscess or generalized peritonitis
0DTJ0	Resection of Appendix, Open Approach	44970	Laparoscopy, surgical, appendectomy
0DTJ4	Resection of Appendix, Percutaneous Endoscopic Approach	44979	Unlisted laparoscopy procedure, appendix
0DTJ7	Resection of Appendix, Via Natural or Artificial Opening		
0DTJ8	Resection of Appendix, Via Natural or Artificial Opening Endoscopic		
0DDJ3	Extraction of Appendix, Percutaneous Approach		
0DDJ4	Extraction of Appendix, Percutaneous Endoscopic Approach		
0DDJ8	Extraction of Appendix, Via Natural or Artificial Opening		
0DCJ0	Extirpation of Matter from Appendix, Open Approach		
0DCJ3	Extirpation of Matter from Appendix, Percutaneous Approach		
0DCJ4	Extirpation of Matter from Appendix, Percutaneous Endoscopic Approach		
0DCJ7	Extirpation of Matter from Appendix, Via Natural or Artificial Opening		
0DCJ8	Extirpation of Matter from Appendix, Via Natural or Artificial Opening Endoscopic		
0D9J0	Drainage of Appendix, Open Approach		
0D9J3	Drainage of Appendix, Percutaneous Approach		
0D9J4	Drainage of Appendix, Percutaneous Endoscopic Approach		
0D9J7	Drainage of Appendix, Via Natural or Artificial Opening		
0D9J8	Drainage of Appendix, Via Natural or Artificial Opening Endoscopic		

© 2024 Kulasekere DA et al. *JAMA Network Open.*

eTable 2. ***International Classification of Diseases, Tenth Revision, Clinical Modification*** **(*ICD-10-CM*) Codes for Abdominal Complaints**

ICD-10-CM Code	Description
A04	Other bacterial intestinal infections
A08	Viral and other unspecified intestinal infections
A09	Other gastroenteritis and colitis of infectious and unspecified origin
K29	Gastritis and duodenitis
K52	Other and unspecified noninfective gastroenteritis and colitis
K59	Constipation, unspecified
K65	Peritonitis
R10	Abdominal and pelvic pain
R11	Nausea and vomiting
R19	Other symptoms and signs involving the digestive system and abdomen

© 2024 Kulasekere DA et al. *JAMA Network Open.*

eFigure. Flow Diagram of Patient Sample Construction (2016 and 2017)

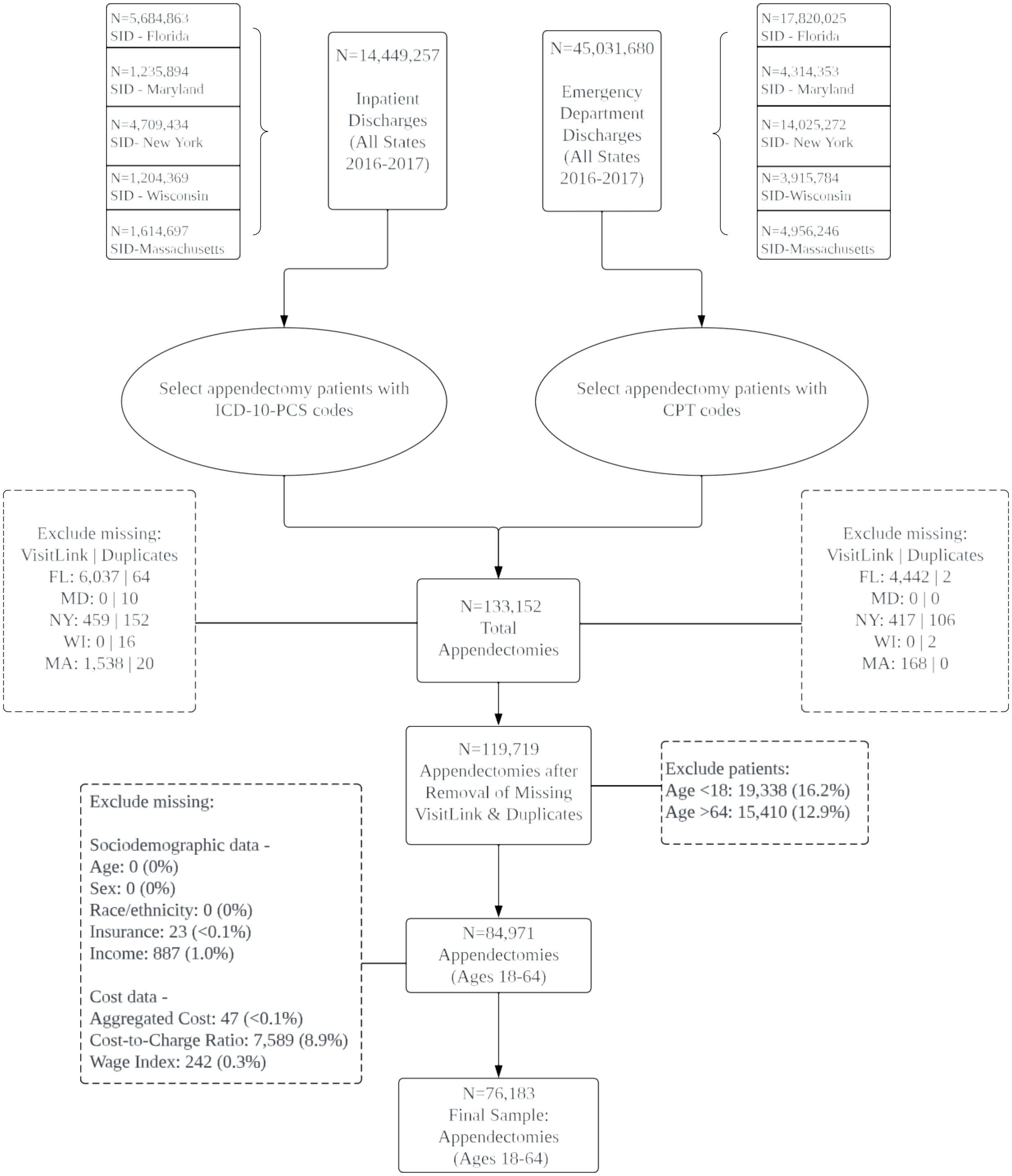

© 2024 Kulasekere DA et al. *JAMA Network Open.*
