# Supplementary material for: Appendicitis Hospitalization Care Costs Among Patients With Delayed Diagnosis of Appendicitis
Source: JAMA Netw Open. 2024 Apr 15;7(4):e246721. doi: 10.1001/jamanetworkopen.2024.6721 (PMC11019393; doi:10.1001/jamanetworkopen.2024.6721)
Supplement: Supplement 2. — Data Sharing Statement [file jamanetwopen-e246721-s002.pdf]

Data Sharing Statement

Kulasekere. Appendicitis Hospitalization Care Costs Among Patients With Delayed Diagnosis of Appendicitis. *JAMA Netw Open*. Published April 15, 2024.
doi:10.1001/jamanetworkopen.2024.6721

Data
Data available: No

Additional Information
Explanation for why data not available: These data are under a specific data use agreement with HCUP
